# Design and functional preliminary investigation of recombinant antigen EgG1Y162–EgG1Y162 against *Echinococcus granulosus*

**DOI:** 10.1515/biol-2022-0558

**Published:** 2023-03-14

**Authors:** Yanxia Zhou, Shangqi Zhao, Yanmin Li, Mingkai Yu, Jia Zheng, Qiaoqiao Gong, Chunbao Cao, Jianbing Ding, Xiaotao Zhou

**Affiliations:** Department of Immunology, Basic Medical College, Xinjiang Medical University, Xinjiang 830011, Urumqi, China; Xinjiang Key Laboratory of Molecular Biology for Endemic Diseases, Xinjiang 830011, Urumqi, China; Department of Anesthesiology, Zhanjiang Central Hospital, Zhanjiang, 524037, Guangdong, China

**Keywords:** *Echinococcus granulosus*, recombinant vaccine EgG1Y162-GGGGSGGG-EgG1Y162, bioinformatics, dendritic cell

## Abstract

In the early stage, our research group cloned *Echinococcus granulosus*-specific antigen, EgG1Y162, from protoscolex and adult worms of *E. granulosus*. In order to enhance the immunogenicity of the vaccine, we prepared a recombinant vaccine by tandemly linking EgG1Y162, splicing the protein and linker at the gene level. This approach is expected to improve the immunogenicity of the vaccine by enhancing the molecular weight of the protein and increasing the antigenic epitopes. Bioinformatics was used to predict the physicochemical properties, transmembrane domain, protein structure, and T-/B-cell antigenic epitope of different recombinant proteins, EgG1Y162-linker-EgG1Y162. Finally, the linker sequence, “GGGGSGGG,” which had the least influence on the migration of recombinant protein T/B epitope and can fold normally in series with EgG1Y162, was selected to design the recombinant vaccine. The plasmid was produced using genetic engineering techniques, and the recombinant protein, EGG1Y162-GGGGSGGG-EgG1Y162, was induced to be expressed and purified. EgG1Y162-GGGGSGGG-EgG1Y162 was identified to be correctly expressed with 100% specificity. Compared with EgG1Y162, EgG1Y162-GGGGSGGG-EgG1Y162 was more likely to promote dendritic cell maturation. EgG1Y162-GGGGSGGG-EgG1Y162 was speculated to have the potential to improve antigen immunogenicity by increasing the molecular weight and antigenic epitope.

## 1 Introduction

Echinococcosis, also known as hydatid disease, is a zoonotic parasitic disease caused by the infection of an intermediate host with *Echinococcosis granulosus* [1]. It has two subtypes, cystic echinococcosis and vesicular echinococcosis. Cystic echinococcosis is more serious and causes high mortality. Cystic echinococcosis is distributed worldwide and common in countries and regions with developed animal husbandry. The current treatment for echinococcosis is still not ideal and has limitations. Therefore, the development of a vaccine is of great interest and is an ideal way to prevent echinococcosis [2,3,4,5]. The selection of specific antigenic targets is the primary task of preparing recombinant vaccines [6,7]. In the study of *E. granulosus* [8], Cao et al. discovered a new gene, *EgG1Y162*, which was sent to the gene bank. In addition, a series of experiments confirmed that the recombinant protein, EgG1Y162, could react with the serum of dogs infected with *E. granulosus*, which indicates that the antigen has high specificity and sensitivity in the immune response of the body. Relevant studies found that specific antibodies can be increased *in vivo* after mice were immunized with EgG1Y162 antigen, which promotes the proliferation of lymphocytes and participates in the cellular and humoral immunity of the body [9]. The prediction and analysis of EgG1Y162 epitope by bioinformatics revealed that this antigen has abundant epitope information, which can enhance the immune response of an organism and induce immune protection [10]. Zhang et al. showed that the recombinant protein EgG1Y162 had good antigenicity [11]. In the present study, recombinant vaccines were prepared by tandemly linking two EgG1Y162 proteins with linker sequences to increase the immunogenicity and immunoreactivity of the vaccine. Three linker sequences, namely, GSGGSG, GGGGSGGG, and GSGGSGGGSGGSGGG, were used to design the recombinant vaccines. The resultant vaccines were compared in terms of physicochemical properties, structure, and antigenic epitopes by bioinformatics method [10], and the most suitable linker sequence was selected. The recombinant protein EgG1Y162-linker-EgG1Y162 was successfully prepared and identified, and the immune effect and mechanism of the recombinant protein were preliminarily explored.

## 2 Materials and methods

### 2.1 Materials

#### 2.1.1 Sequences of EgG1Y162 and EgG1Y162–linker–EgG1Y162 proteins

The amino acid sequence of the EgG1Y162 protein, which has a total length of 120 aa, was stored in GenBank with accession number AB458259. The recombinant proteins, EgG1Y162-GSGGSG-EgG1Y162, EgG1Y162-GGGGSGGG-EgG1Y162, and EgG1Y162-GSGGSGGGSGGSGGG-EgG1Y162, had total lengths of 246, 248, and 255 aa, respectively.

EgG1Y162:

VDPELMAKLTKELKTTLPEHFRWIHVGSRSLELGWNATGLANLHADHIKLTANLYTTYVTFKYRNVPIERQKLTLEGLKPSTFYEVVQAFKGGSQVFKYTGFIRTLAPGEDGADRASGF

EgG1Y162-GSGGSG-EgG1Y162:

VDPELMAKLTKELKTTLPEHFRWIHVGSRSLELGWNATGLANLHADHIKLTANLYTTYVTFKYRNVPIERQKLTLEGLKPSTFYEVVVQAFKGGSQVFKYTGFIRTLAPGEDGADRASGFGSGVDPELMAKLTKELKTTLPEHFRWIHVGSRSLELGWNATGLANLHADHIKLTANLYTTYVTFKYRNVPIERQKLTLEGLKPSTFYEVVVQAFKGGSQVFKYTGFIRTLAPGEDGADRASGF

EgG1Y162-GGGGSGGG-EgG1Y162:

VDPELMAKLTKELKTTLPEHFRWIHVGSRSLELGWNATGLANLHADHIKLTANLYTTYVTFKYRNVPIERQKLTLEGLKPSTFYEVVVQAFKGGSQVFKYTGFIRTLAPGEDGADRASGFGGGGSGGGVDPELMAKLTKELKTTLPEHFRWIHVGSRSLELGWNATGLANLHADHIKLTANLYTTYVTFKYRNVPIERQKLTLEGLKPSTFYEVVVQAFKGGSQVFKYTGFIRTLAPGEDGADRASGF

EgG1Y162-GSGGSGGGSGGSGGG-EgG1Y162:

VDPELMAKLTKELKTTLPEHFRWIHVGSRSLELGWNATGLANLHADHIKLTANLYTTYVTFKYRNVPIERQKLTLEGLKPSTFYEVVVQAFKGGSQVFKYTGFIRTLAPGEDGADRASGFGSGGSGGSGGSGGGVDPELMAKLTKELKTTLPEHFRWIHVGSRSLELGWNATGLANLHADHIKLTANLYTTYVTFKYRNVPIERQKLTLEGLKPSTFYEVVQAFKGGSQVFKYTGFIRTLAPGEDGADRASGF

#### 2.1.2 Bioinformatics software

The following softwares were used in the bioinformatics analysis: ProtParam (http://web.expasy.org/protparam/), Transmembrane Helices Hidden Markov Model (TMHMM) server (http:/www.cbs.dtu.dk/services/TMHMM-2.0/) [5], Self-Optimized Prediction Method with Alignment (SOPMA, https://npsa-prabi.ibcp.fr/cgibin/npsa_automat.pl? page = npsa_sopma.html), BepiPred1.0 server (http://www.cbs.dtu.dk/services/BepiPred-1.0/), Immune Epitope Database (IEDB, http://tools.iedb.org/main/bcell/, http://tools.iedb.org/mhcii/), SVMTriP (http://sysbio.unl.edu/SVMTriP/prediction.php), Propred (http://imed.med.ucm.es/Tools/rankpep.html), and SYFPEITHI (http://www.syfpeithi.de/bin/mhcserver.dll/epitopeprediction).

#### 2.1.3 Plasmids and reagents

The glycerol expression bacteriophage EgG1Y162 was kept in our laboratory. Plasmid EgG1Y162-GGGGGSGGG-EgG1Y162 was constructed and synthesized by Shanghai Biotechnology Company, China. *Escherichia coli* BL21 (DE3) receptor cells were obtained from Shanghai Weidi Biotechnology Co., Ltd (China). Lysogeny broth (LB) liquid and solid media were purchased from Shanghai Biotechnology Co. (China). Agarose was purchased from Invitrogen, USA. Plasmid extraction kit was purchased from Beijing Tiangen Co. (China). Polymerase chain reaction (PCR) kits, Q cutting enzymes (EcoRI and SalI), and DNA Maker2000 were purchased from Dalian TaKaRa Co. (Kyoto, Japan). Pre-stained Protein Maker was purchased from Thermo Fisher Scientific (Massachusetts, US). Isopropyl β-D-thiogalactoside (IPTG) and sodium dodecyl sulfate–polyacrylamide gel electrophoresis (SDS–PAGE) gel preparation kit were purchased from Solarbio (Beijing, China). Polyvinylidene difluoride membrane (PVDF) was purchased from GE Healthcore (Little Chalfont, UK). His-Tag Mouse antibody was purchased from Cell Signaling Technology (BD biosciences, Franklin Lakes, US). Goat anti-mouse IgG-horseradish peroxidase (HRP) was purchased from Absin Biotechnology Co. (Shanghai, China). Goat anti-human IgG-HRP was purchased from Beijing Bioss Biotechnology Co. (China). High-sensitivity enhanced chemiluminescence (ECL) test kit was purchased from Comwin Biotech Co., Ltd (Beijing, China). HisTrap purification columns were purchased from General Electric, USA. RPMI 1640 medium, fetal bovine serum (BI), and double antibodies were purchased from Hyclone. Erythrocyte lysate was purchased from Beijing Solarbio Technology Co. (China). RmIL-4 and rmGM-CSF were purchased from PeproTech (New Jersey, US). Fluorescein isothiocyanate (FITC)-coupled anti-HIS antibody was purchased from Beijing Bioss Biotechnology Co. (China). Recombinant cytokine lysis dilution kit was purchased from Hangzhou Unitech Biotechnology Co. (China).

#### 2.1.4 Experimental animals

Specific pathogen-free C57 mice (6–8 weeks, 20 ± 2 g) were purchased from the Experimental Animal Center of Xinjiang Medical University (License No：SCXK(Xin)2016-0003).


**Ethical approval:** The research related to animal use has been complied with all the relevant national regulationsand institutional policies for the care and use of animals.

### 2.2 Methods

#### 2.2.1 Bioinformatics prediction method

ProtParam was initially used to analyze the physicochemical properties of each recombinant protein, including protein molecular mass, theoretical isoelectric point, extinction coefficient, and other theoretical properties. Then, the TMHMM server was used to predict and analyze the transmembrane region of each recombinant protein, and SOPMA was used in the secondary structure prediction of each recombinant protein with the transmembrane region to improve the reliability of the pretreatment. The 3D model of recombinant proteins was established using the online software, I-TASSER. BepiPred1.0 server and SVMTriP were used to analyze the possible dominant linear epitopes of each recombinant protein on B cells. Propred, IEDB, and SYFPEITHI were used to predict the T-cell antigenic epitopes of each recombinant protein. HLA-DRB1*0701 was selected as the parameter. The epitope with the highest SYFPEITHI score (the higher the score, the higher the probability that the sequence will be the dominant epitope) and lowest IEDB percentage rank (the lower the percent rank, the higher the probability of the sequence being the dominant epitope) was determined. Multiple results were compared, and the prediction results of secondary structures were referred to exclude the structures that could not easily form epitopes. Based on the predicted results of B-cell and T-cell epitopes, the length and location of each predicted epitope were evaluated. The sequences that were too short to form epitopes were excluded, and peptides with high multiple prediction repetition rates were selected as the possible dominant T/B combined epitopes of recombinant proteins.

#### 2.2.2 Identification of prokaryotic expression plasmid and induction expression, purification, and identification of recombinant protein

The synthesized plasmid, pET30a-EgG1Y162-GGGGSGGG-EgG1Y162, was identified by double digestion with EcoRI and SalI. The correctly identified plasmid was transformed into the host bacterium, *E. coli* BL21 (DE3). The single clone was selected and inoculated in 20 mL of LB liquid medium containing 30 μg/mL kanamycin and incubated overnight on a shaking table at 220 rpm at 37°C. The next day, the overnight culture was inoculated at a ratio of 1:50 in LB liquid medium containing 30 μg/mL kanamycin to expand the culture. The bacterial culture solution was cultured on a shaking table at 220 rpm at 37°C until the optical density of the bacterial solution was 0.6–0.8 and induced for 6 h with a final IPTG concentration of 0.5 mmol/L at 28°C. The target protein in the supernatant was obtained and purified, and the expression of recombinant protein was identified by Western blot. His-Tag antibodies (Cell Signaling Techonology, Beverly, MA, US, Cat. N.#9991) were used for Western blot detection.

#### 2.2.3 Western blot analysis of recombinant protein expression

The purified proteins were electrotransferred to the PVDF membrane after SDS–PAGE. Then, the membrane was closed with 5% skimmed milk powder at room temperature for 1 h and repeatedly washed three times with 1× TBST for 15 min each time. Finally, the primary antibody was added and incubated overnight at 4°C on a shaking table. Cysticercosis mouse serum and normal mouse serum were used as primary antibodies which were diluted at a concentration of 1:150 and incubated overnight. The antibodies were discarded the next day, and the PVDF membrane was washed three times with 1× TBST for 15 min each time. Goat anti-mouse IgG-HRP (Absin, Shanghai, China, Cat. N.abs20039)(1:3000 dilution) was added, incubated for 2 h at room temperature, and washed three times with 1× TBST for 15 min each time. ECL was added for color development, and the results were observed. Specificity was calculated using the formula:

\text{Specificity}=\text{True negative}{/}(\text{True negative}+\text{False negative})\times 100 \% .]

#### 2.2.4 Maturation of mouse bone marrow-derived dendritic cells (DCs) after 24 h stimulation with recombinant protein

The mice were sacrificed by cervical dislocation. The tibia and femur were removed under aseptic conditions, and the muscle tissue was stripped. Appropriate PBS was absorbed with a syringe and inserted into the epiphysis to clean the bone cavity and obtain bone marrow cells. The cells were filtered through a filter and centrifuged, and the supernatant was discarded. The cells were added with an appropriate amount of erythrocyte lysate, mixed, and placed in a refrigerator at 4°C for 10 min, and centrifuged for 5 min at room temperature, and the supernatant was discarded. The bone marrow cells were suspended in a complete culture medium, inoculated into six-well plates with rmGM-CSF and rmIL-4, incubated in a CO_2_ incubator with half volume of fluid change every day, and added with the corresponding cytokines until day 7 to obtain immature dendritic cells (imDCs). EgG1Y162 (final concentration 500 ng/mL) and EgG1Y162-GGGGSGGG-EgG1Y162 (final concentration 500 ng/mL) were co-cultured with imDC for 24 h, and the cell suspension was collected. Then, flow cytometric identification was performed. The cells (1 × 10^6^) were placed in a flow tube, added with PBS (2 mL), and centrifuged at 1,000 rpm for 5 min. The supernatant was discarded, and 50 μL 1× PBS containing 1 μL CD86 + antibody, 1 μL CD11c + antibody, 1 μL CD45 + antibody, and 1 μL I-Ab antibody was added and incubated at 4°C for 30 min in the dark. The cells were washed by adding 1 mL 1× PBS and centrifuged at 1,000 rpm and 4°C for 5 min. The cells were repeatedly cleaned with PBS and then added with 300 μL PBS to resuscitate the cells. The percentage of mature DC was detected within 4 h.

### 2.3 Statistics

All data were shown by Mean Value ± Standard Deviation (\bar{x}\pm s)] . The statistical analysis was done by SPSS19.0 software, and the data of multiple groups were analyzed by one-way variance analysis. When the P value was less than 0.05, it indicated a significant difference. The software GraphdPad Prism5.0 was used for drawing the map.

## 3 Results

### 3.1 Bioinformatics analysis showed that “GGGGSGGG” was connected to the EgG1Y162 protein as the linker sequence and none of the epitopes shifted

#### 3.1.1 Comparison of the physicochemical properties of recombinant proteins and prediction of transmembrane regions

**Figure 1:**
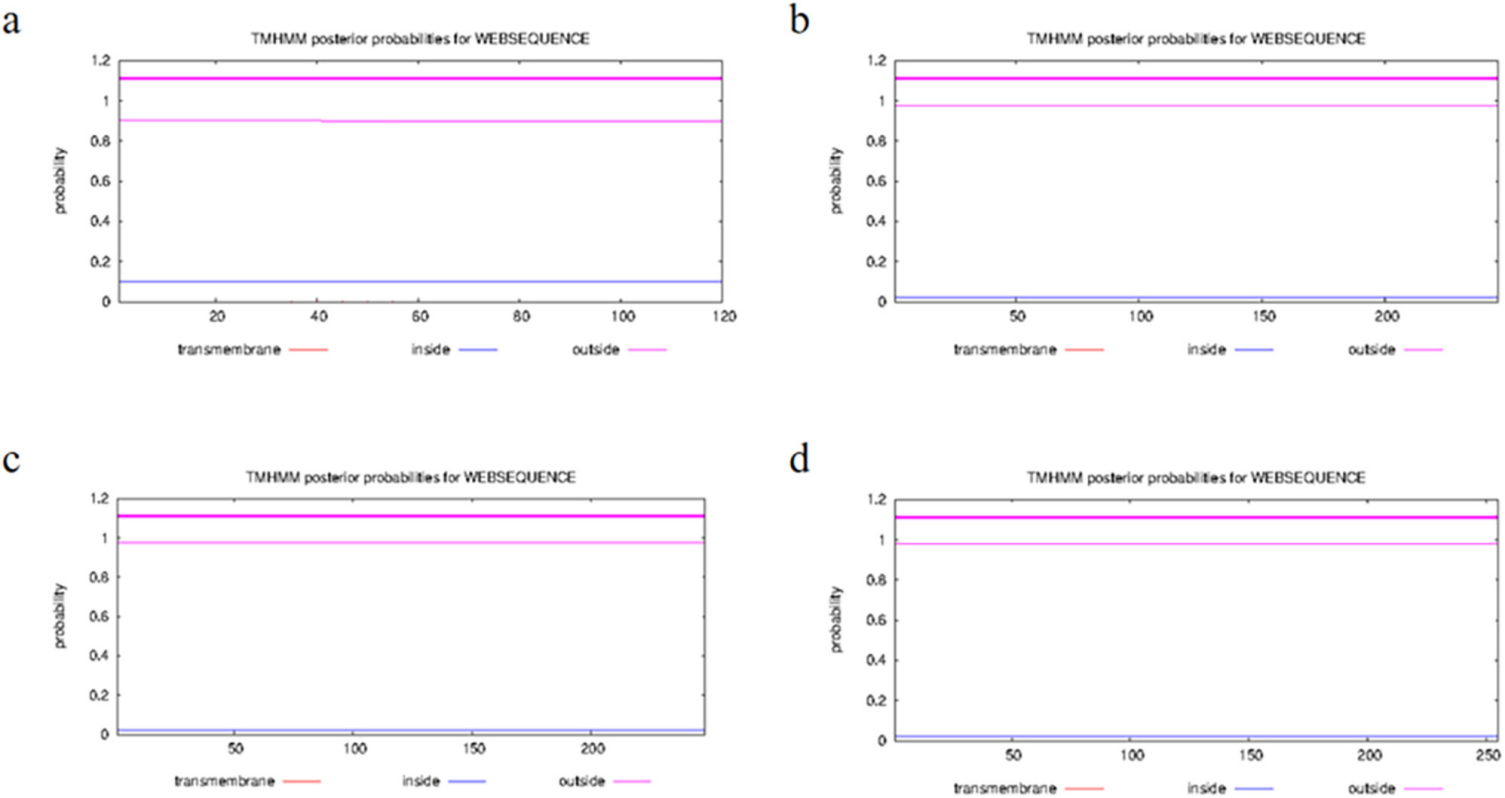
Transmembrane domain of each recombinant protein. (a) EgG1Y162; (b) EGG1Y162-GSGGSG-EGG1Y162; (c) EGG1Y162-GGGGSGGG-EGG1Y162; (d) EGG1y162-GSGGSGGSGGG-EGG1Y162.

The physicochemical properties of the recombinant proteins were analyzed by using ProtParam (http://web.expasy.org/protparam/). The result showed that the EgG1Y162 protein is composed of 120 aa and has a molecular mass of 13515.49 Ka, a theoretical isoelectric point (pI) value of 9.22; a chemical molecular formula of C_619_H_960_N_164_O_174_S_1_, an extinction coefficient of 18,450, an instability index of 29.98 (>40 means unstable protein, and < 40 is stable protein), and a grand average of hydropathicity index (GRAVY) of −0.263 (the overall GRAVY range is between −2 and 2; negative values indicate hydrophilic proteins). The EgG1Y162-GSGGSG-EgG1Y162 protein is composed of 246 aa and has a molecular mass of 27415.32 Da, theoretical pI value of 9.34, a chemical formula of C_1252_H_1940_N_334_O_355_S_2_, an extinction coefficient of 36,900, an instability index of 30.04, and a GRAVY of −0.270. The EgG1Y162-GGGGSGGG-EgG1Y162 protein is composed of 248 aa and has a protein molecular mass of 27499.40 Da, a theoretical pI value of 9.34, a chemical formula of C_1255_H_1944_N_336_O_356_S_2_, an extinction coefficient of 36,900, an instability index of 31.87, and a GRAVY of −0.269. The EgG1Y162-GSGGSG-EgG1Y162 protein is composed of 246 aa and has a molecular mass of 27415.32 Da, a theoretical pI value of 9.34, a chemical formula of C_1252_H_1940_N_334_O_355_S_2_, an extinction coefficient of 36,900, an instability index of 30.04, and a GRAVY of −0.270. The EgG1Y162-GSGGSGGGSGGSGGG-EgG1Y162 protein is composed of 255 aa and has a molecular mass of 27988.84 Da, a theoretical pI value of 9.34, chemical formula of C_1272_H_1971_N_343_O_366_S_2_, an extinction coefficient of 36,900, an instability index of 31.75, and a GRAVY of −0.278. The TMHMM server was used to analyze the transmembrane region of each recombinant protein. The transmembrane protein regions were greater than 1, which indicates that each recombinant protein is an extracellular protein that can be fully contacted by antigen-presenting cells and initiate a strong immune response from T and B cells. The results are shown in Figure 1.

#### 3.1.2 Prediction of the secondary structures of each recombinant protein

**Figure 2:**
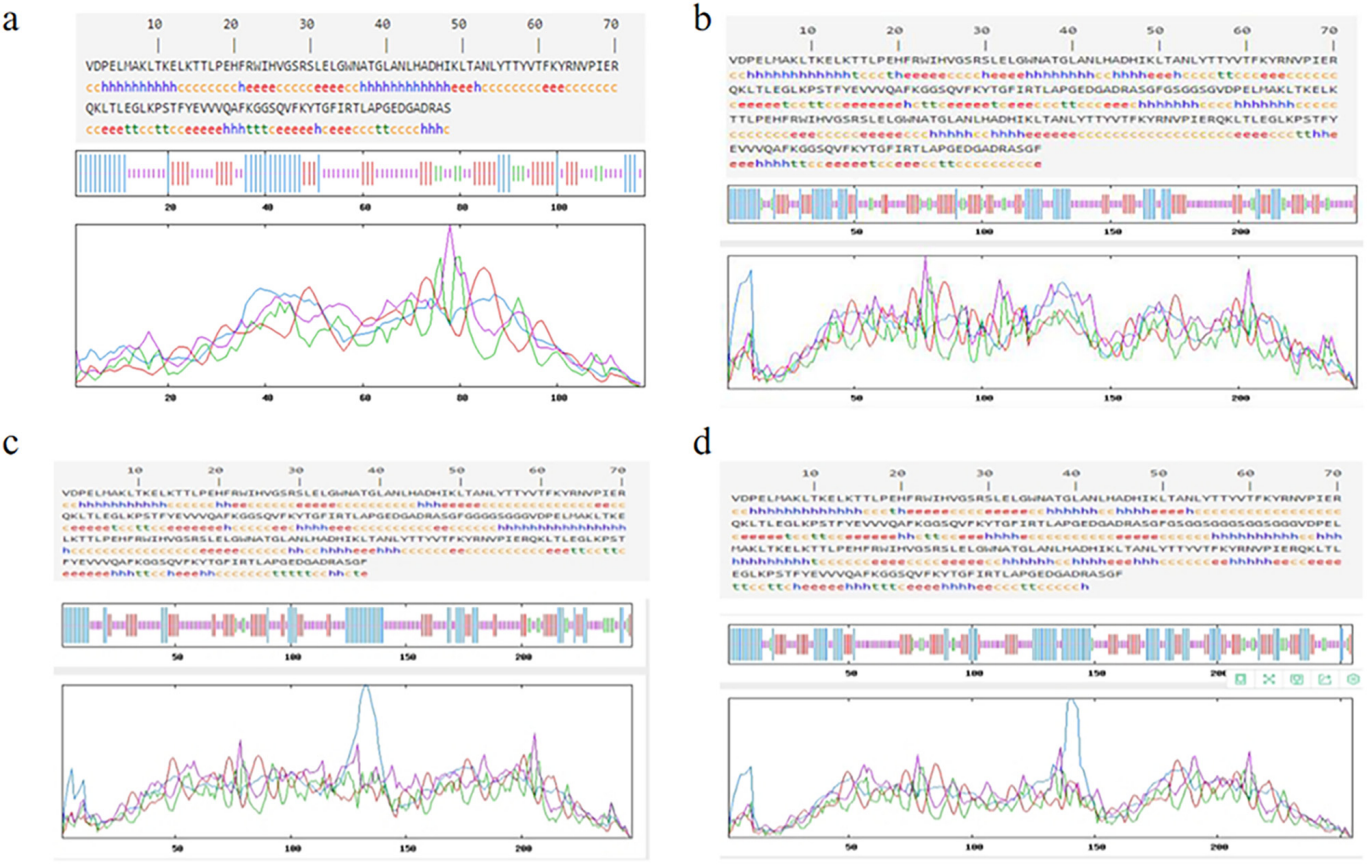
SOMPA analysis of the secondary structures of recombinant proteins: (a) EgG1Y162; (b) EgG1Y162-GSGGSG-EgG1Y162; (c) EgG1Y162-GGGGSGGG-EgG1Y162; (d) EgG1Y162-GSGGSGGGSGSGGG-EgG1Y162.

The secondary structures of each recombinant protein were predicted and analyzed by SOPMA online software. The predicted results demonstrate the secondary structures of each recombinant protein and the proportions of various structural domains present in the protein, including alpha helix, extended strand, beta turn, and random coil, as shown in Figure 2.

#### 3.1.3 Analysis of the T-/B-cell antigenic epitopes of recombinant proteins

BepiPred1.0 Server [12], SVMTriP, IEBD, and SYFPEITHI software were used to predict the possible dominant antigenic epitopes of EgG1Y162 and the EgG1Y162-linker-EgG1Y162 proteins on T/B cells. The predicted results showed that EgG1Y162 has three B-cell antigen epitopes, which are located at 19–28, 60–75, and 108–117 aa (Table 1), and three dominant T-cell antigen epitopes, which are located at 7–21, 57–69, and 77–111 aa (Table 2). Recombinant protein EgG1Y162-GSGGSG-EgG1Y162 has six B-cell antigen epitopes, which are located at 19–26, 60–68, 108–130, 147–154, 186–205, and 235–242 aa (Table 3), and six dominant T-cell antigen epitopes, which are located at 7–37, 45–69, 95–111, 133–160, 171–195, and 221–237 aa (Table 4). Recombinant protein EgG1Y162-GGGGSGGG-EgG1Y162 has six B-cell antigen epitopes, which are located at 19–28, 60–79, 108–132, 149–166, 188–207, and 236–244 aa (Table 5), and six dominant T-cell epitopes, which are located at 10–37, 45–69, 95–111, 135–165, 173–198, and 220–237 aa (Table 6). Recombinant protein EgG1Y162-GSGGSGGGSGGSGGG-EgG1Y162 has six B-cell antigen epitopes, which are located at 19–26, 57–70, 108–139, 157–178, 195–205, and 243–252 aa (Table 7), and six dominant T-cell epitopes, which are located at 8–21, 45–69, 95–111, 155–172, 180–207, and 230–246 aa (Table 8). The T-/B-cell antigenic epitopes of EgG1Y162 and EgG1Y162-linker-EgG1Y162 recombinant proteins are shown in Table 9. EgG1Y162-GGSGGG-EgG1Y162 had less epitope migration compared with EgG1Y162-GSGGSGGGSGGSGGG-EgG1Y162 and EgG1Y162-GSGGSG-EgG1Y162. Therefore, the recombinant protein EgG1Y162-linker-EgG1Y162 whose linker sequence was “GGGGSGGG” was selected for verification.

**Table 1:** Prediction results of the B-cell epitope of recombinant protein EgG1Y162

Bepi Pred1.0		SVMTriP		IEDB	
Position	Sequence	Position	Sequence	Position	Sequence
108–120	APGEDGADRASGF	19–38	EHFRWIHVGSRSLELGWNAT	14–28	KTTLPEHFRWIHVGS
		60–79	TFKYRNVPIERQKLTLEGLK	55–75	YTTYVTFKYRNVPIERQKLTL
				105–117	RTLAPGEDGADRA

**Table 2:** Prediction results of the T-cell epitope of recombinant protein EgG1Y162

SYFPEITHI			IEDB		
Position	Sequence	Score	Position	Sequence	Rank
32–46	ELGWNATGLANLHAD	26	22–36	RWIHVGSRSLELGWN	0.90
2–16	DPELMAKLTKELKTT	24	23–37	WIHVGSRSLELGWNA	1.70
95–109	SQVFKYTGFIRTLAP	18	42–56	NLHADHIKLTANLYT	3.60
18–32	PEHFRWIHVGSRSLE	16	57–71	TYVTFKYRNVPIERQ	11.00
80–94	PSTFYEVVVQAFKGG	16	58–72	YVTFKYRNVPIERQK	11.00
4–18	ELMAKLTKELKTTLP	14	7–21	AKLTKELKTTLPEHF	12.00
47–61	HIKLTANLYTTYVTF	14	77–91	GLKPSTFYEVVVQAF	12.00
62–76	KYRNVPIERQKLTLE	14	92–106	KGGSQVFKYTGFIRT	12.00
77–91	GLKPSTFYEVVVQAF	14	97–111	VFKYTGFIRTLAPGE	12.00
104–118	IRTLAPGEDGADRAS	14			

**Table 3:** Prediction results of the B-cell epitopes of recombinant protein EgG1Y162-GSGGSG-EgG1Y162

Bepi Pred1.0		SVMTriP		IEDB	
Position	Sequence	Position	Sequence	Position	Sequence
108–130	APGEDGADRASGFGSGGSGVDPE	19–38	EHFRWIHVGSRSLELGWNAT	15–26	TTLPEHFRWIHV
234–246	APGEDGADRASGF	145–164	EHFRWIHVGSRSLELGWNAT	57–68	TYVTFKYRNVPI
		60–79	TFKYRNVPIERQKLTLEGLK	107–145	LAPGEDGADRASGFGSGGSGVDPELMAKLTKELKTTLPE
		186–205	TFKYRNVPIERQKLTLEGLK	147–154	FRWIHVGS
				181–206	YTTYVTFKYRNVPIERQKLTLEGLKP
				235–242	PGEDGADR

**Table 4:** Prediction results of the T-cell epitope of recombinant protein EgG1Y162-GSGGSG-EgG1Y162

Propred			SYFPEITHI			IEDB		
Position	Sequence	Score	Position	Sequence	Score	Position	Sequence	Score
173–181	HIKLTANLY	13.661	32–46	ELGWNATGLANLHAD	26	23–37	WIHVGSRSLELGWNA	1.70
47–55	HIKLTANLY	13.661	146–160	HFRWIHVGSRSLELG	26	149–163	WIHVGSRSLELGWNA	1.70
170–178	HADHIKLTA	12.667	158–172	ELGWNATGLANLHAD	26	42–56	NLHADHIKLTANLYT	3.60
44–52	HADHIKLTA	12.667	2–16	DPELMAKLTKELKTT	26	168–182	NLHADHIKLTANLYT	3.60
			128–142	DPELMAKLTKELKTT	24	10–24	TKELKTTLPEHFRWI	8.30
			10–24	TKELKTTLPEHFRWI	24	136–150	TKELKTTLPEHFRWI	8.30
			22–36	RWIHVGSRSLELGWN	24	57–71	TYVTFKYRNVPIERQ	11.00
			45–59	ADHIKLTANLYTTYV	22	183–197	TYVTFKYRNVPIERQ	11.00
			75–89	LEGLKPSTFYEVVVQ	22	58–72	YVTFKYRNVPIERQK	11.00
			171–185	ADHIKLTANLYTTYV	22	184–198	YVTFKYRNVPIERQK	11.00
			201–215	LEGLKPSTFYEVVVQ	22	7–21	AKLTKELKTTLPEHF	12.00
			55–69	YTTYVTFKYRNVPIE	18	133–147	AKLTKELKTTLPEHF	12.00
			95–109	SQVFKYTGFIRTLAP	18	92–106	KGGSQVFKYTGFIRT	12.00
			100–114	YTGFIRTLAPGEDGA	18	77–91	GLKPSTFYEVVVQAF	12.00
			117–131	ASGFGSGGSGVDPEL	18	203–217	GLKPSTFYEVVVQAF	12.00
			181–195	YTTYVTFKYRNVPIE	18	218–232	KGGSQVFKYTGFIRT	12.00
			221–235	SQVFKYTGFIRTLAP	18	97–111	VFKYTGFIRTLAPGE	12.00
			226–240	YTGFIRTLAPGEDGA	18	223–237	VFKYTGFIRTLAPGE	12.00

**Table 5:** Prediction results of the B-cell epitope of recombinant protein EgG1Y162-GGGGSGGG-EgG1Y162

Bepi Pred1.0		SVMTriP		IEDB	
Position	Sequence	Position	Sequence	Position	Sequence
108–132	APGEDGADRASGFGGGGSGGGVDPE	19–38	EHFRWIHVGSRSLELGWNAT	14–28	KTTLPEHFRWIHVGS
236–248	APGEDGADRASGF	147–166	EHFRWIHVGSRSLELGWNAT	38–43	TGLANL
		60–79	TFKYRNVPIERQKLTLEGLK	56–80	TTYVTFKYRNVPIERQKLTLEGLKP
		188–207	TFKYRNVPIERQKLTLEGLK	108–147	APGEDGADRASGFGGGGSGGGVDPELMAKLTKELKTTLPE
				149–154	FRWIHV
				166–171	TGLANL
				183–208	YTTYVTFKYRNVPIERQKLTLEGLKP
				221–230	GGSQVFKYTG
				236–244	APGEDGADR

**Table 6:** Prediction results of the T-cell epitope of recombinant protein EgG1Y162-GGGGSGGG-EgG1Y162

Propred			SYFPEITHI			IEDB		
Position	Sequence	Score	Position	Sequence	Score	Position	Sequence	Score
175–183	HIKLTANLY	13.661	32–46	ELGWNATGLANLHAD	26	21–35	FRWIHVGSRSLELGW	0.70
47–55	HIKLTANLY	13.661	148–162	HFRWIHVGSRSLELG	26	150–164	RWIHVGSRSLELGWN	0.90
172–180	HADHIKLTA	12.667	160–174	ELGWNATGLANLHAD	26	23–37	WIHVGSRSLELGWNA	1.70
44–52	HADHIKLTA	12.667	2–16	DPELMAKLTKELKTT	24	151–165	WIHVGSRSLELGWNA	1.70
			130–144	DPELMAKLTKELKTT	24	42–56	NLHADHIKLTANLYT	3.60
			10–24	TKELKTTLPEHFRWI	22	170–184	NLHADHIKLTANLYT	3.60
			22–36	RWIHVGSRSLELGWN	22	56–70	TTYVTFKYRNVPIER	11.00
			45–59	ADHIKLTANLYTTYV	22	184–198	TTYVTFKYRNVPIER	11.00
			75–89	LEGLKPSTFYEVVVQ	22	186–200	YVTFKYRNVPIERQK	11.00
			138–152	TKELKTTLPEHFRWI	22	7–21	AKLTKELKTTLPEHF	12.00
			173–187	ADHIKLTANLYTTYV	22	135–149	AKLTKELKTTLPEHF	12.00
			203–217	LEGLKPSTFYEVVVQ	22	77–91	GLKPSTFYEVVVQAF	12.00
			55–69	YTTYVTFKYRNVPIE	18	205–219	GLKPSTFYEVVVQAF	12.00
			95–109	SQVFKYTGFIRTLAP	18	92–106	KGGSQVFKYTGFIRT	12.00
			100–114	YTGFIRTLAPGEDGA	18	220–234	KGGSQVFKYTGFIRT	12.00
			117–131	ASGFGGGGSGGGVDP	18	97–111	VFKYTGFIRTLAPGE	12.00
			183–197	YTTYVTFKYRNVPIE	18	225–235	VFKYTGFIRTLAPGE	12.00
			223–237	SQVFKYTGFIRTLAP	18			
			228–242	YTGFIRTLAPGEDGA	18			

**Table 7:** Prediction results of the B-cell epitope of recombinant protein EgG1Y162-GSGGSGGGSGGSGGG-EgG1Y162

Bepi Pred1.0		KSVMTriP		IEDB	
Position	Sequence	Position	Sequence	position	Sequence
108–139	APGEDGADRASGFGSGGSGGGSGGSGGGVDPE	19–38	EHFRWIHVGSRSLELGWNAT	10–26	TKELKTTLPEHFRWIHV
243–255	APGEDGADRASGF	154–173	EHFRWIHVGSRSLELGWNAT	38–43	TGLANL
		60–79	TFKYRNVPIERQKLTLEGLK	57–70	TYVTFKYRNVPIER
		195–214	TFKYRNVPIERQKLTLEGLK	107–154	LAPGEDGADRASGFGSGGSGGGSGGSGGGVDPELMAKLTKELKTTLPE
				157–161	RWIHV
				173–178	TGLANL
				192–205	TYVTFKYRNVPIER
				241–252	TLAPGEDGADRA

**Table 8:** Prediction results of the T-cell epitope of recombinant protein EgG1Y162-GSGGSGGGSGGSGGG-EgG1Y162

Propred			SYFPEITHI			IEDB		
Position	Sequence	Score	Position	Sequence	Score	Position	Sequence	Score
182–190	HIKLTANLY	13.661	32–46	ELGWNATGLANLHAD	26	154–168	EHFRWIHVGSRSLEL	0.64
47–55	HIKLTANLY	13.661	155–169	HFRWIHVGSRSLELG	26	22–36	RWIHVGSRSLELGWN	0.90
179–187	HADHIKLTA	12.667	2–16	DPELMAKLTKELKTT	24	23–37	WIHVGSRSLELGWNA	1.70
44–52	HADHIKLTA	12.667	137–151	DPELMAKLTKELKTT	24	158–172	WIHVGSRSLELGWNA	1.70
			10–24	TKELKTTLPEHFRWI	22	42–56	NLHADHIKLTANLYT	3.60
			45–59	ADHIKLTANLYTTYV	22	177–191	NLHADHIKLTANLYT	3.60
			75–89	ADHIKLTANLYTTYV	22	8–22	KLTKELKTTLPEHFR	11.00
			180–194	ADHIKLTANLYTTYV	22	56–70	TTYVTFKYRNVPIER	11.00
			210–224	LEGLKPSTFYEVVVQ	22	191–205	TTYVTFKYRNVPIER	11.00
			165–179	SLELGWNATGLANLH	20	58–72	YVTFKYRNVPIERQK	11.00
			55–69	YTTYVTFKYRNVPIE	18	193–207	YVTFKYRNVPIERQK	11.00
			95–109	SQVFKYTGFIRTLAP	18	7–21	AKLTKELKTTLPEHF	12.00
			100–114	YTGFIRTLAPGEDGA	18	142–156	AKLTKELKTTLPEHF	12.00
			117–131	ASGFGSGGSGGGSGG	18	77–91	GLKPSTFYEVVVQAF	12.00
			190–204	YTTYVTFKYRNVPIE	18	212–226	GLKPSTFYEVVVQAF	12.00
			230–244	SQVFKYTGFIRTLAP	18	92–106	KGGSQVFKYTGFIRT	12.00
			235–249	YTGFIRTLAPGEDGA	18	227–241	KGGSQVFKYTGFIRT	12.00
						230–244	SQVFKYTGFIRTLAP	12.00
						97–111	VFKYTGFIRTLAPGE	12.00
						232–246	VFKYTGFIRTLAPGE	12.00

**Table 9:** Prediction of T-/B-cell antigen co-epitopes of each recombinant protein

Recombinant protein	Position		T/B combined epitope
	B-Cell antigen epitopes	T-Cell antigen epitopes	
EgG1Y162	19–28	7–18	AKLTKE*LKTTLP**EHFRWI*** **HVGS**
	60–75	47–71	*HIKLTANLYTT**YVTFKYRNVPIERQ*** **KLTL**
	108–117	95–118	*SQVFKYTGFIRT**LAPGEDGADRA**S*
EgG1Y162-GSGGSG-EgG1Y162	19–26	7–37	*AKLTKELKTTLP**EHFRWIHV**GSRSLELGWNA*
	60–68	45–69	*ADHIKLTANLYTTYV**TFKYRNVPI**E*
	108–130	95–111	*SQVFKYTGFIRTL**APGE*** **DGADRASGFGSGGSGVDPE**
	147–154	133–160	*AKLTKELKTTLPEH**FRWIHVG**SRSLELG*
	186–205	171–195	*ADHIKLTANLYTTYV**TFKYRNVPIE*** **RQKLTLEGLK**
	235–242	221–237	*SQVFKYTGFIRTLA**PGE*** **DGADR**
EgG1Y162-GGGGSGGG-EgG1Y162	19–28	10–37	*TKELKTTLP**EHFRWIHVGS**RSLELGWNA*
	60–79	45–69	*ADHIKLTANLYTTYV**TFKYRNVPIE*** **RQKLTLEGLK**
	108–132	95–111	*SQV**FKYT*** **GFIRTLAPGEDGADRASGFGGG**
	149–166	135–165	*AKLTKELKTTLPEH**FRWIHVGSRSLELGWNA*** **T**
	188–207	173–198	*ADHIKLTANLYTTYV**TFKYRNVPIER*** **QKLTLEGLK**
	236–244	220–237	*KGGSQVFKYTGFIRTE**AP*** **GEDGADR**
EgG1Y162-GSGGSGGGSGGSGGG-EgG1Y162	19–26	8–21	*KLTKELKTTLP**EHF*** **RWIHV**
	57–70	45–69	*ADHIKLTANLYT**TYVTFKYRNVPIE*** **R**
	108–139	95–111	*SQVFKYTGFIRTL**APGE*** **DGADRASGFGSG**
	157–178	155–172	*HF**RWIHVGSRSLELGWNA*** **TGLANL**
	195–205	180–207	*ADHIKLTANLYTTYV**TFKYRNVPIER**QK*
	243–252	230–246	*SQVFKYTGFIRTL**APGE*** **DGADRA**

Note: T-cell epitopes are in italics, and B-cell epitopes are underlined.

#### 3.1.4 Tertiary structure prediction

**Figure 3:**
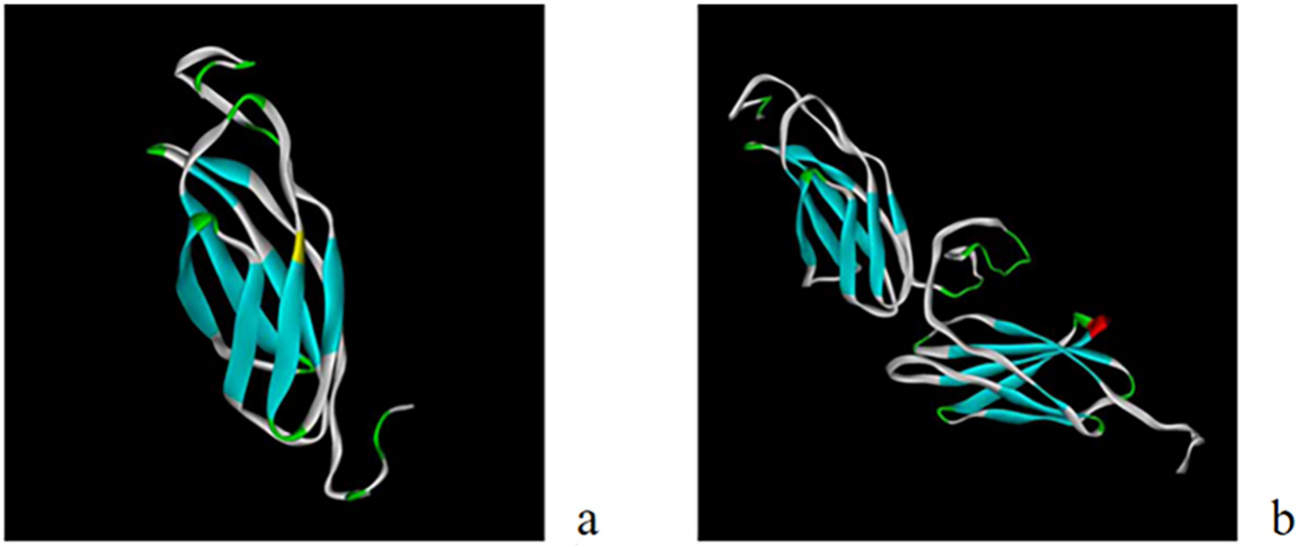
Prediction of the tertiary structure of recombinant protein: (a) EgG1Y162; (b) EgG1Y162-GGGGSGGG-EgG1Y162.

I-TASSER [13,14] was used to predict the tertiary structures of EgG1Y162 and EgG1Y162-GGGGSGGG-EgG1Y162. In the prediction of the tertiary structure of EgG1Y162 (Figure 3a), the C-score was −2.25 (C-score ranges from −5 to 2, and a higher score shows a model with higher confidence), the template modeling (TM) score was 0.45 ± 0.14 (TM > 0.5 shows a correct topological model, and TM < 0.17 suggests a randomly similar model), and the root mean square deviation (RMSD) was 10.9 ± 4.6 Å. In the prediction of the tertiary structure of EgG1Y162-GGGGSGGG-EgG1Y162 (Figure 3b), the C-score was −1.55; the TM score was 0.52 ± 0.15, and the RMSD was 7.6 ± 4.3 Å. The results showed that in EgG1Y162-GGGGSGGG-EgG1Y162, the two EgG1Y162 in series can be expressed normally.

### 3.2 EgG1Y162-GGGGSGGG-EgG1Y162 promoted DC maturation by increasing antigenic epitopes, presumably with possible strong immunogenicity

#### 3.2.1 The recombinant plasmid was correctly constructed and the prokaryotic expression of recombinant protein was correctly induced

**Figure 4:**
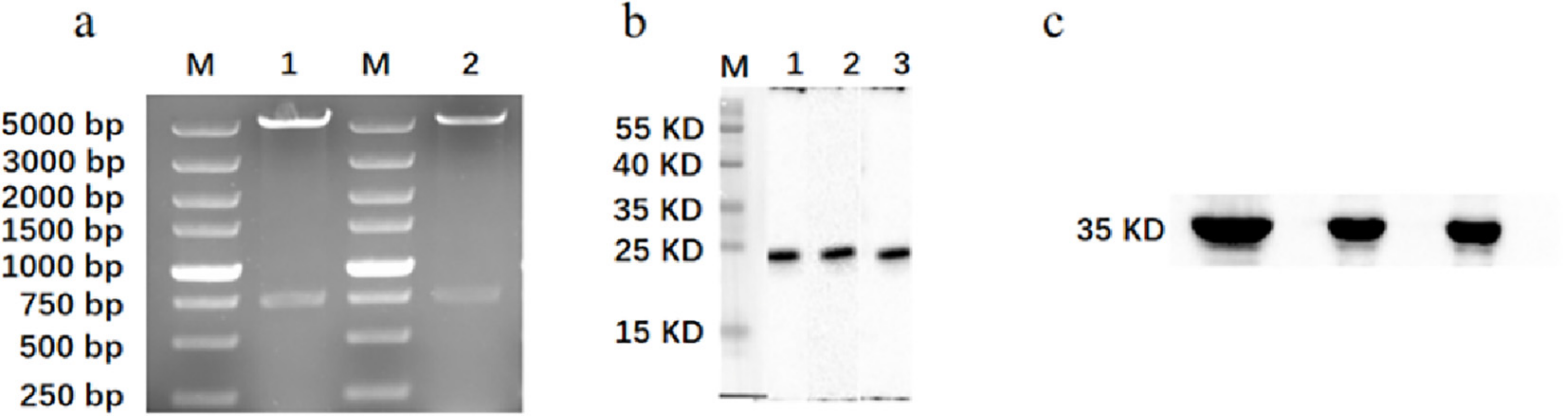
Identification of recombinant plasmid pET30a-EgG1Y162-GGGGSGGG-EgG1Y162 digestion and protein purification: (a) identification of recombinant plasmid pET30a-EgG1Y162-GGGGSGGG-EgG1Y162 digestion. M: DL5000; 1 and 2: Recombinant plasmids digested by EcoRI and SalI, respectively. (b) Western blot analysis of purified EgG1Y162. M: protein molecular quality standard; 1–3: EgG1Y162. (c) Western blot analysis of purified HIS-EgG1Y162-GGGGSGGG-EgG1Y162.

The recombinant plasmid, pET30a-EgG1Y162-GGGGSGGG-EgG1Y162, was double digested by EcoRI and SalI pairs, and the target fragments with sizes of about 5,400 and 756 bp, which were consistent with the expected size, were obtained by 1% agarose gel electrophoresis (Figure 4a). The induced expression of purified recombinant proteins, HIS-EgG1Y162 and HIS-EgG1Y162-GGGGGSGGG-EgG1Y162, showed distinct bands at 20.5 (Figure 4b) and 35 kDa (Figure 4c), respectively, which were in accordance with the expected results.

#### 3.2.2 Specificity of EgG1Y162-GGGGGSGGG-EgG1Y162

**Figure 5:**
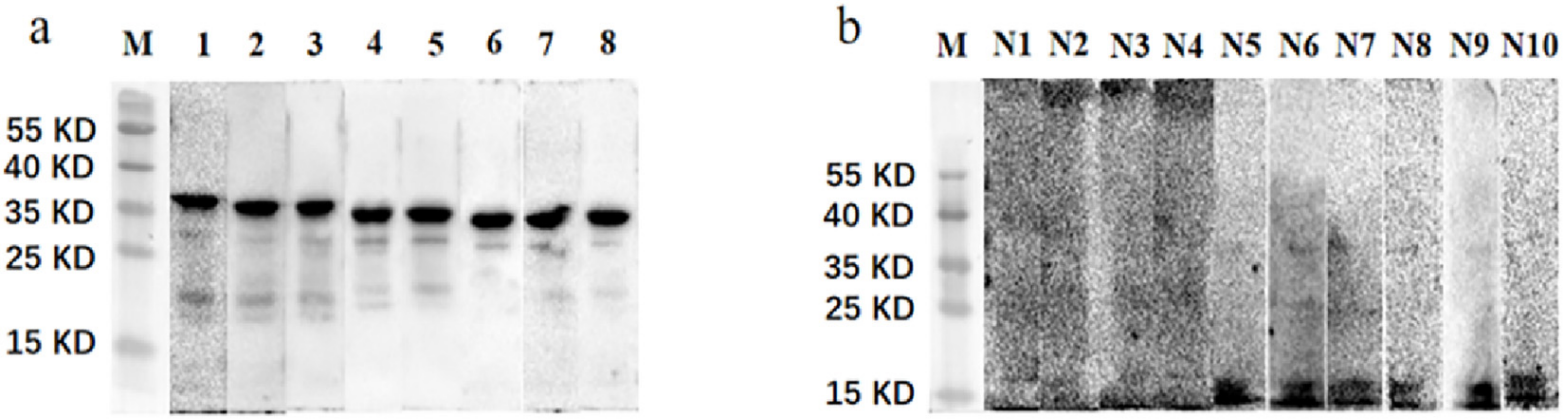
Western blot identification of mouse sera. M: protein molecular quality standard; (a) 1–8: sera of cystic hydatid mouse; (b) 1–10: normal mouse sera.

The serum of 10 normal mice was analyzed by Western blot. None of the serum of the normal mice showed obvious reaction bands at about 35 kDa, whereas obvious reaction bands were found in the serum of 8 mice infected with *E. granulosus* at about 35 kDa of the target band. The calculated specificity was 100% as shown in Figure 5.

#### 3.2.3 EgG1Y162-GGGGSGGG-EgG1Y162 promoted the maturation of the mouse bone morrow-derived DCs

**Figure 6:**
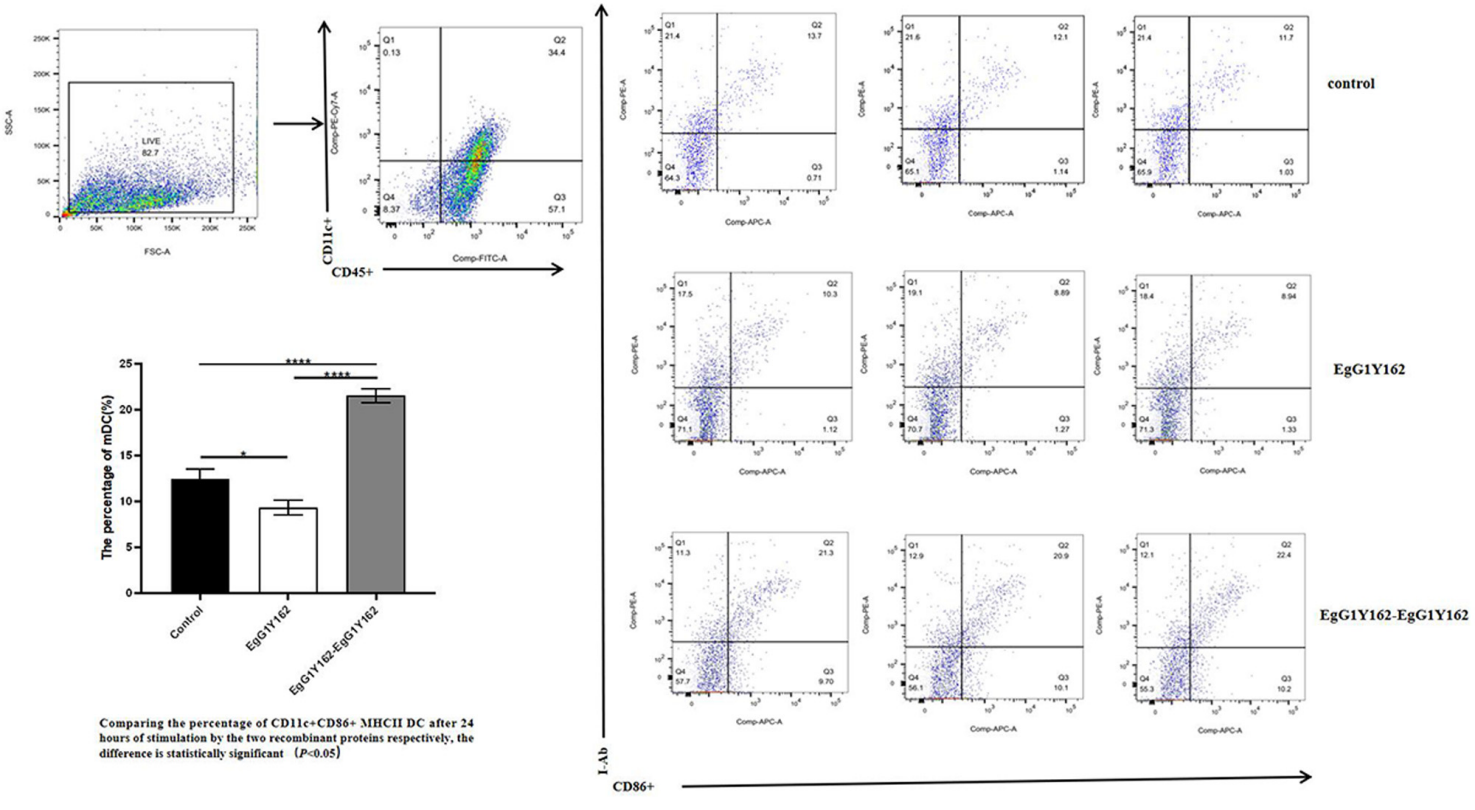
Flow cytometry analysis of the percentage of mature DCs after EgG1Y162 and EgG1Y162-GGGGSGGG-EgG1Y162 stimulation for 24 h.

After the mouse bone marrow-derived DCs were stimulated with recombinant protein for 24 h, the percentage of mature DCs in the EgG1Y162-GGGGSGGG-EgG1Y162-stimulated group (21.533 ± 0.777%) was significantly higher than that of the EgG1Y162-stimulated group (9.37 ± 0.800%) as shown in Figure 6 (*t* = 18.883, *P* < 0.01).

## 4 Discussion

Echinococcosis is an endemic and natural zoonotic disease caused by *E. granulosus* infection in intermediate livestock, especially in the pastoral areas of Northwest China [15]. It has a severe impact on human health, and the extent of the disease gradually increases, which will seriously restrict economic development [16,17]. The current treatment method for hydatid disease is still relatively limited, and the effect of surgery or medical treatment is not ideal [18,19]. Therefore, vaccination is an effective way to prevent the infection of hydatid disease, and the development of vaccine has become a hot spot of current research [20]. Our research group previously demonstrated that the antigen EgG1Y162 of *E. granulosus* has good immunogenicity and can induce the body to produce an effective immune response for immune protection [21]. The immunogenicity of the antigen has a great relationship with its state and time of existence in the body. An antigen with a larger molecular weight and a more stable protein state is stronger, exists in the organism for a longer period of time, has a stronger immunogenicity, and can produce a stronger immune response; thus, it can enable the organism to obtain better protection. In the present study, we tried to cascade antigen EgG1Y162 by selecting an appropriate linker sequence and increasing the molecular weight of the recombinant protein to achieve a better immune response and improve the immunogenicity of the antigen.

With the development of computer bioinformatics technology, bioinformatics has been increasingly used to predict protein structures [22]. Therefore, in this study, the structures of EgG1Y162-linker-EgG1Y162 recombinant proteins with different linker sequences were predicted by bioinformatics methods. The results showed that the physicochemical properties, hydrophilicity, and stability of EgG1Y162 did not change after the incorporation of a series of long, medium, and short linker sequences. We further examined whether the T-/B-cell antigen epitope shifted in the three EgG1Y162-linker-EgG1Y162 recombinant proteins to select the best linker sequence. Therefore, SYFPEITHI [23], IEDB [24], BepiPred [25], and SVMTriP [26] were used to predict the epitopes of the recombinant proteins. HLA-DRB1*0701 was selected as the parameter. Ten results were compared to determine which epitope had the highest SYFPEITHI score (the higher the score, the more likely the sequence is to be the dominant epitope) and the lowest IEDB percentile rank (the lower the percentile rank, the more likely the sequence is to be the dominant epitope). Based on the results of secondary structures, the structures that could not form an epitope were excluded. In EgG1Y162-GSGGSG-EgG1Y162, the T- and B-cell antigen epitopes did not change or shift. We found through data integration that the T-/B-cell antigen epitopes of EgG1Y162-GGGGSGGG-EgG1Y162 are located at 10–37 (equivalent to 10–37 aa on EgG1Y162, “TKELKTTLPEHFRWIHVGSRSLELGWNA”), 45–79 (equivalent to 45–79 aa on EgG1Y162, “ADHIKLTANLYTTYVTFKYRNVPIERQKLTLEGLK”), 95–132 (equivalent to 95–120 aa on EgG1Y162, “SQVFKYTGFIRTLAPGEDGADRASGFGGG”), 135–166 (equivalent to 7–38 aa on EgG1Y162, “AKLTKELKTTLPEHFRWIHVGSRSLELGWNAT”), and 173–207 (equivalent to 45–79 aa on EgG1Y162, “ADHIKLTANLYTTYVTFKYRNVPIERQKLTLEGLK”), and 220–244 aa (equivalent to 92–116 aa on EgG1Y162, “KGGSQVFKYTGFIRTEAPGEDGADR”). The analysis showed that among the three recombinant proteins, the epitope of EgG1Y162-GGGGSGGG-EgG1Y162 almost had no offset. This result also suggests that EgG1Y162 could be connected in series by linker sequence “GGGGSGGG” to increase the molecular weight of the protein without affecting the original epitope.

Tertiary structure prediction was performed on EgG1Y162 and EgG1Y162-GGGGSGGG-EgG1Y162 to determine whether linker sequence “GGGGSGGG” affects the expression of proteins on both sides. Tertiary structure prediction is performed through three methods: homology modeling, line string method, and ab initio prediction method. I-TASSER [14,27] online prediction server uses two sets of algorithms, namely, homology modeling and line string method, to model and predict the protein’s tertiary structure; its accuracy and reliability are much higher than those of Swiss Model and PHYRE, and its 3D model is based on the multi-line LOMETS and iterative TASSER. Predictive models were derived to match the database of BioLiP protein functions, and the first one provided is the most reliable template parameter [28]. In the tertiary structure prediction of EgG1Y162, the C-score was −1.55, the TM score was 0.52 ± 0.15, and the RMSD was 7.6 ± 4.3 Å. In the tertiary structure prediction of EgG1Y162-GGGGSGGG-EgG1Y162, the C-score was −2.25, the TM score was 0.45 ± 0.14, and the RMSD was 10.9 ± 4.6 Å. The prediction results of the 3D structures of the two proteins showed that the contiguous proteins can be correctly folded with the addition of the linker sequence “GGGGSGGG,” which further confirmed the feasibility of selecting the linker sequence “GGGGSGGG.” The recombinant antigen is linked to the same antigen to increase the specific epitope, so as to cause a strong immune response. There are relevant studies linking different antigens to prepare a multi-epitope vaccine, which also has significant immune effects and good safety, providing a new idea for the follow-up experiments of this study [29,30,31].

A large number of recombinant proteins need to be induced to conduct animal immunization experiments. The research group explored the induced expression conditions of the recombinant protein and found that the IPTG concentration of recombinant plasmid pET30a-EgG1Y162 was 0.2 mmol/L at 28°C, and the induced expression of recombinant protein EgG1Y162 was the highest in the supernatant after 6 h of induction. The protein expression level of recombinant plasmid pET30a-EgG1Y162-GGGGSGGG-EgG1Y162 was the highest in the supernatant at 28°C, the final IPTG concentration was 0.5 mmol/L, and the induction lasted for 6 h. In the Western blot method, we applied the His-Tag tag antibody for the initial identification of the proteins. The clear bands at approximately 20.5 and 35 kDa indicated that the EgG1Y162 and EgG1Y162-GGGGSGGG-EgG1Y162 were successfully induced. In further serological validation, the recombinant protein EgG1Y162-GGGGSGGG-EgG1Y162 antigenicity was analyzed in the sera of diseased mice and normal mice, and the specificity of the protein was found to be 100% in both cases.

The antigen presentation process of vaccine entry into the organism starts with dedicated APCs [24,25]. APCs are the first gate to trigger the immune response after vaccine entry into the organism [32]. DCs are the most functional APCs in the body. Under normal conditions, most DCs in the body are in the immature stage, and imDCs have a strong capacity for antigen endocytosis and processing [33]. After a series of processes, such as antigen uptake and inflammatory factor activation, DCs change from immature to mature, and mature DCs highly express antigen-presenting molecules, MHC class II molecules, and co-stimulatory molecules, such as CD54, CD40, CD80, and CD86, which initiate MHC-II class-restricted CD4 + Th2 responses, promote the antibody production of B cells and cell-mediated immunity, and play a good immunoprotective effect [34,35,36]. Purified HIS-EgG1Y162 and HIS-EgG1Y162-GGGGSGGG-EgG1Y162 were separately co-cultured with the DCs obtained by *in vitro* induction. Compared with HIS-EgG1Y162, DCs were more likely to mature under HIS-EgG1Y162-GGGGSGGG-EgG1Y162 stimulation. This finding suggests that EgG1Y162-GGGGSGGG-EgG1Y162 stimulation promoted the maturation of mouse bone marrow-derived DCs and thus enhanced the immune response. We preliminarily speculated that the recombinant vaccine, EgG1Y162-GGGGSGGG-EgG1Y162, had improved immunogenicity because of the increased molecular weight and repeating T-/B-cell epitope.

In summary, we predicted and screened the best linker sequence by bioinformatics prediction and successfully induced the expression of the recombinant vaccine, EgG1Y162-GGGGSGGG-EgG1Y162. The results of serological identification in mice showed that the recombinant vaccine had good specificity. The antigen epitopes contained in the recombinant vaccine were increased by increasing the molecular weight of the recombinant vaccine to promote the maturation of DC and improve the immunogenicity of the vaccine. The design and preparation of recombinant protein EgG1Y162-GGGGGSGGG-EgG1Y162 provide a new idea for the optimization and improvement of the vaccine against encapsulated diseases. The development of a safe and effective hydatid vaccine is only the first step toward eliminating hydatid, and we need to anticipate implementation strategies and acceptance. Therefore, a comprehensive evaluation of the reliability, safety, and benefits of a candidate vaccine needs to be carried out before it can be put into clinical trials.
